# Mitochondrial Calcium Uniporter MCU Supports Cytoplasmic Ca^2+^ Oscillations, Store-Operated Ca^2+^ Entry and Ca^2+^-Dependent Gene Expression in Response to Receptor Stimulation

**DOI:** 10.1371/journal.pone.0101188

**Published:** 2014-07-08

**Authors:** Krishna Samanta, Sophie Douglas, Anant B. Parekh

**Affiliations:** Department of Physiology, Anatomy and Genetics, University of Oxford, Oxford, United Kingdom; Vanderbilt University Medical Center, United States of America

## Abstract

Ca^2+^ flux into mitochondria is an important regulator of cytoplasmic Ca^2+^ signals, energy production and cell death pathways. Ca^2+^ uptake can occur through the recently discovered mitochondrial uniporter channel (MCU) but whether the MCU is involved in shaping Ca^2+^ signals and downstream responses to physiological levels of receptor stimulation is unknown. Here, we show that modest stimulation of leukotriene receptors with the pro-inflammatory signal LTC_4_ evokes a series of cytoplasmic Ca^2+^ oscillations that are rapidly and faithfully propagated into mitochondrial matrix. Knockdown of MCU or mitochondrial depolarisation, to reduce the driving force for Ca^2+^ entry into the matrix, prevents the mitochondrial Ca^2+^ rise and accelerates run down of the oscillations. The loss of cytoplasmic Ca^2+^ oscillations appeared to be a consequence of enhanced Ca^2+^-dependent inactivation of InsP_3_ receptors, which arose from the loss of mitochondrial Ca^2+^ buffering. Ca^2+^ dependent gene expression in response to leukotriene receptor activation was suppressed following knockdown of the MCU. In addition to buffering Ca^2+^ release, mitochondria also sequestrated Ca^2+^ entry through store-operated Ca^2+^ channels and this too was prevented following loss of MCU. MCU is therefore an important regulator of physiological pulses of cytoplasmic Ca^2+^.

## Introduction

Mitochondrial Ca^2+^ import plays a fundamental role in cell physiology through shaping the spatial and temporal profile of intracellular Ca^2+^ signals, stimulating ATP production and regulating cell survival [1], [2]. The outer mitochondrial membrane is freely permeable to Ca^2+^ but the inner membrane is not. Ca^2+^ uptake across the latter is accomplished through the mitochondrial Ca^2+^ uniporter (MCU). Although this transporter has been known to exist for several decades, only recently has it been identified at a molecular level. MCU comprises a membrane-spanning 40 kDa protein that forms a low conductance Ca^2+^-selective channel pore [3], [4], [5]. Important regulators of MCU activity have been discovered including MICU1 [6] and MICU2 [7], MCUR1 [8] and EMRE [9]. Ca^2+^ transporters that extrude Ca^2+^ from the matrix have also been characterised recently, including the mitochondrial Na^+^-Ca^2+^ exchanger [10] and Letm1 [11], a Ca^2+^-H^+^ exchanger.

Ca^2+^ uptake by MCU is determined by both the large voltage across the inner membrane that results from proton pumping by the respiratory chain, and the Ca^2+^ concentration gradient between the cytoplasm and matrix [12], [13]. Knockdown of MCU using siRNA-based strategies significantly reduced the rise in mitochondrial matrix Ca^2+^ that followed a cytoplasmic Ca^2+^ increase [3], [4]. However, these previous investigations on MCU have tended to use high non-physiological concentrations of agonist, raising the question whether MCU contributes to physiological levels of Ca^2+^ signalling.

Stimulation of cell-surface receptors that couple to phospholipase C generates the second messengers InsP_3_ and diacylglycerol [14]. Modest levels of receptor activation, which are thought to mirror physiological levels of receptor occupancy, result in repetitive cytoplasmic Ca^2+^ oscillations that arise from regenerative Ca^2+^ release from InsP_3_-sensitive Ca^2+^ stores followed by Ca^2+^ entry through store-operated Ca^2+^ release-activated Ca^2+^ (CRAC) channels, which refills the stores in readiness for the next oscillatory cycle. Information is encoded in the amplitude, frequency and spatial profile of the oscillation [15]. In mast cells, activation of cysteinyl leukotriene type I (cysLT1) receptors with the physiological agonist leukotriene C_4_ evokes a series of Ca^2+^ oscillations [16] and local Ca^2+^ entry through CRAC channels during the oscillatory responses activates the transcription factors NFAT [17] and c-fos [16], which interact to regulate expression of chemokines and cytokines that shape the subsequent local inflammatory response.

In this study, we have investigated whether MCU regulates the pattern of oscillatory Ca^2+^ signals and subsequent activation of gene expression following cysLT1 receptor stimulation. We find that MCU is essential for supporting these responses, reinforcing its role as a key regulator of physiological Ca^2+^ signals. We also find that MCU, by buffering Ca^2+^ influx, is important for sustaining CRAC channel activity.

## Results

### Ca^2+^ oscillations run down rapidly after MCU knockdown

**Figure 1:**
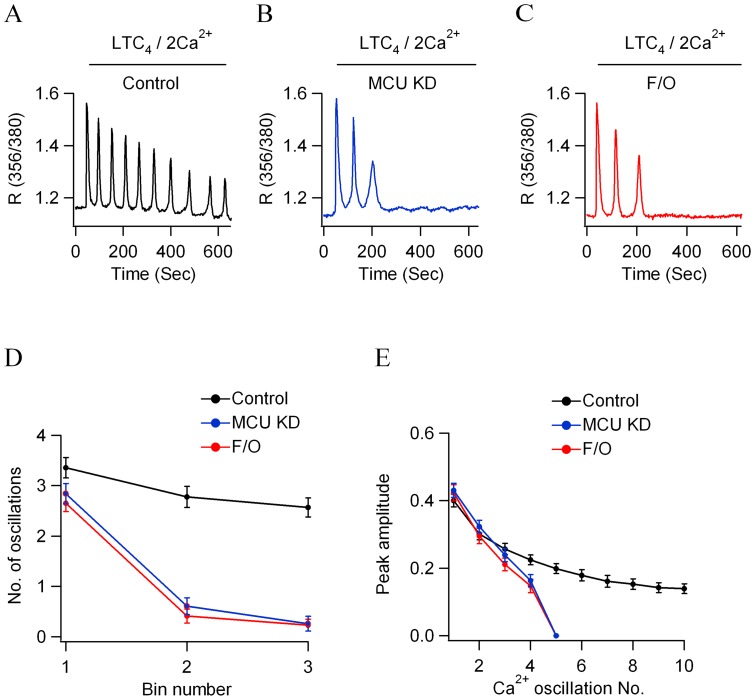
Cytoplasmic Ca^2+^ oscillations run down quickly after MCU knockdown. A, Control recording to LTC_4_ in 2 mM external Ca^2+^. B, Response to LTC_4_ after MCU knockdown. C, Mitochondrial depolarisation with FCCP (5 µM) accelerates run down of oscillations. D, Aggregate data showing the number of oscillations in 200 seconds recording bins from several experiments are compared (each point is the average of between 36 and 51 cells). E, The size of each oscillation is plotted against Ca^2+^ oscillation number for the conditions shown (each point is the average of between 35 and 50 cells).

Stimulation of native receptors in the mast cell line RBL-1 with a submaximal concentration (160 nM) of LTC_4_ evoked a series of cytoplasmic Ca^2+^ oscillations (Figure 1A) that decreased gradually in number (Figure 1D) and size (Figure 1E) due to receptor desensitization [18]. Knockdown of MCU significantly altered the pattern of response. Ca^2+^ oscillations now ran down very quickly (Figure 1B), disappearing within 300 seconds (Figure 1D). The amplitude of each spike following MCU knockdown also declined markedly (Figure 1E). Similar results were obtained when mitochondria were depolarised with FCCP (5 µM), a protonophore that collapses the mitochondrial potential and thereby reduces the electrical gradient for Ca^2+^ uptake via the MCU. In the presence of FCCP, Ca^2+^ oscillations ran down rapidly (Figure 1C), and at a rate and extent similar to that seen after knockdown of MCU (Figure 1D, E).

### Mitochondrial depolarisation correlates with run down of Ca^2+^ oscillations

**Figure 2:**
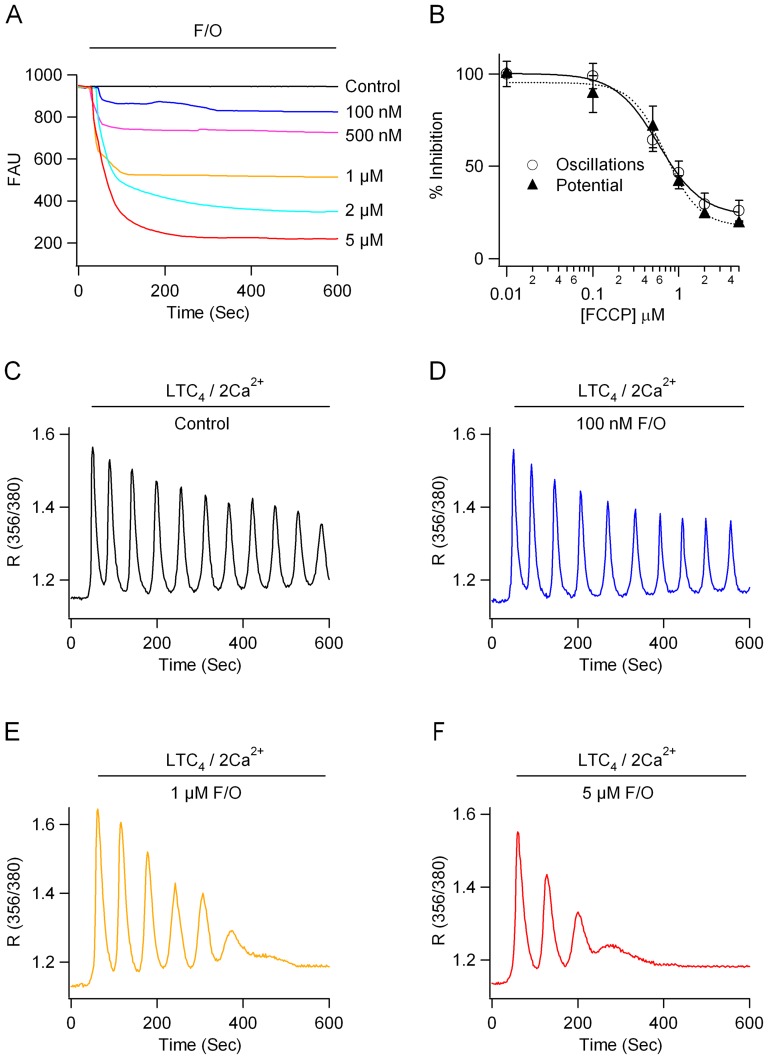
Graded relationship between mitochondrial depolarisation and run down of Ca^2+^ oscillations. A, Increasing the concentration of FCCP leads to increased mitochondrial depolarisation. B, The graph compares the effects of different concentrations of FCCP on mitochondrial membrane potential and the number of Ca^2+^ oscillations evoked by LTC_4_ over a 600 seconds recording period. C, Control recording to LTC_4_. D-F, Effects of increasing FCCP concentration of oscillatory Ca^2+^ signals. Cells from C-F were all from the same cell preparation and were used on the same day.

To see whether mitochondrial depolarisation correlated with run down of the Ca^2+^ oscillations, we compared the effects of FCCP on both mitochondrial membrane potential and the number of Ca^2+^ oscillations that were produced over a 600 seconds recording period. Increasing the concentration of FCCP led to a progressive depolarisation of the mitochondrial membrane potential, measured with TMRE (Figure 2A, B), as well as a decrease in number of Ca^2+^ oscillations (Figure 2C-F). Dose-response curves showing the effects of different concentrations of FCCP on mitochondrial potential and oscillatory number are compared in Figure 2B. Both responses were graded with the concentration of FCCP and showed similar dose-dependencies.

### Mitochondrial matrix Ca^2+^ oscillates in response to physiological stimulation

**Figure 3:**
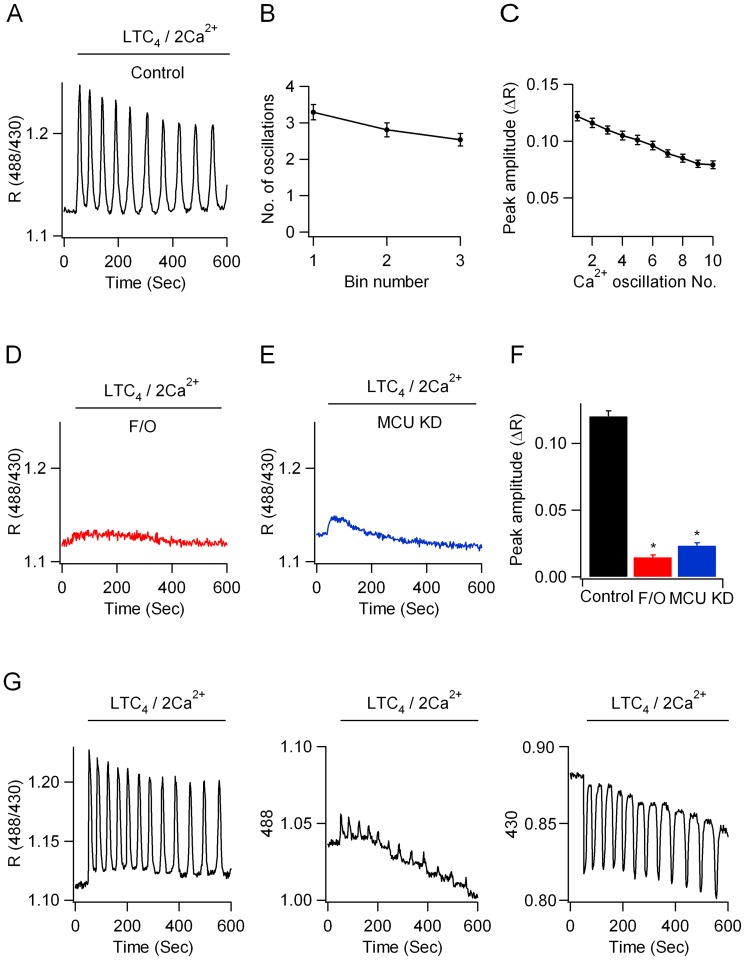
Leukotriene receptor stimulation induces oscillatory Ca^2+^ signals in the mitochondrial matrix. A, Oscillatory Ca^2+^ response to LTC_4_, measured using the ratiometric pericam. B, The number of oscillations per 200 seconds bin are compared for the conditions shown. Each point is the mean of between 16 and 27 cells. C, The amplitude of each oscillation is compared for the conditions shown. D, Mitochondrial depolarisation with FCCP prevents the matrix rise in Ca^2+^. E, Knockdown of MCU prevents matrix Ca^2+^ rise to LTC_4_. F, Aggregate data are summarised for the conditions shown. Each bar is the average of between 12 and 17 cells. G, Single wavelength pericam (488 nm and 430 nm) recordings are shown along with the corresponding ratio.

If mitochondria buffer physiological pulses of cytoplasmic Ca^2+^ through MCU, then matrix Ca^2+^ should rise following challenge with LTC_4_ in an MCU-dependent manner. To test this, we measured mitochondrial Ca^2+^ by expressing the genetically encoded ratiometric pericam that targets to the mitochondrial matrix [19], [20]. Stimulation with LTC_4_ elicited a series of repetitive Ca^2+^ oscillations in matrix Ca^2+^ (Figure 3A) that closely mimicked the cytoplasmic Ca^2+^ oscillations in number and frequency (Figures 3B and 3C; Figure 1). Targeted ratiometric pericam measured mitochondrial Ca^2+^ because the matrix Ca^2+^ rise to LTC_4_ was suppressed by pre-treatment with FCCP (Figure 3D, 3F). Knockdown of MCU suppressed the rise in mitochondrial Ca^2+^ following cysLT1 receptor activation (Figure 3E, F). After MCU knockdown, the initial Ca^2+^ rise was significantly reduced (Figure 3F) and no cell (76/76 cells) showed any subsequent rise in matrix Ca^2+^ following the small initial response.

Ratiometric pericam is sensitive to pH at excitation wavelengths close to 480 nm [19] and we were concerned that the mitochondrial fluorescent signals reflected changes in matrix pH rather than matrix Ca^2+^. Pericam is only weakly sensitive to pH over the wavelengths 415–430 nm. Stimulation with LTC_4_ still evoked oscillatory changes at 430 nm, demonstrating that the pericam was indeed measuring matrix Ca^2+^ under our conditions (Figure 3G).

### MCU is required to sustain regenerative Ca^2+^ release

**Figure 4:**
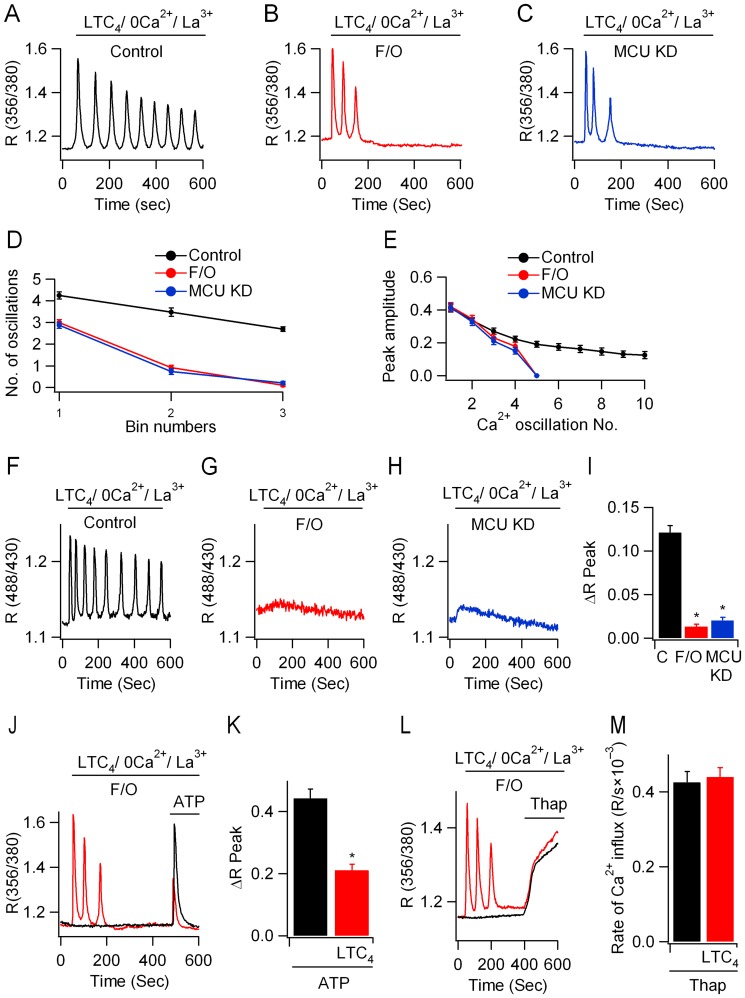
Regenerative Ca^2+^ release in the absence of Ca^2+^ influx is regulated by mitochondrial Ca^2+^ uptake. A, LTC_4_ evokes repetitive Ca^2+^ oscillations in the presence of 0 mM Ca^2+^ external solution supplemented with 1 mM La^3+^. B, The oscillations run down quickly after mitochondrial depolarisation. C, The Ca^2+^ oscillations run down quickly after knockdown of MCU. D, Aggregate data comparing the number of oscillations in each 200 seconds bin from several experiments are compared. E, As in panel D, but the amplitude of each oscillation is compared. F, Oscillatory Ca^2+^ signals are seen in the matrix in response to LTC_4_ in 0 mM Ca^2+^/1 mM La^3+^. G, Matrix Ca^2+^ response is prevented by FCCP. H, Knockdown of MCU also suppresses the matrix Ca^2+^ rise in response to LTC_4_ challenge. I, Aggregate data from several experiments are compared. Each bar is the average of between 11 and 18 cells. J, Ca^2+^ release evoked by P2Y receptor activation is reduced by pre-exposure to LTC_4_. K, Aggregate data from several cells are compared. ATP bar represents 27 cells and LTC_4_/ATP group is 34 cells. L, Ca^2+^ release to thapsigargin is unaffected by prior stimulation with LTC_4_. M, Aggregate data measuring the rate of rise of cytoplasmic Ca^2+^ following thapsigargin application (as in panel L) are compared. Thap bar represents data from 11 cells, and LTC_4_/thap 14 cells.

Ca^2+^ oscillations to LTC_4_ are generated by InsP_3_-dependent Ca^2+^ release but store-operated Ca^2+^ influx is required to replenish the stores with Ca^2+^ in readiness for the next Ca^2+^ release phase [21], [22]. We separated these components by generating Ca^2+^ oscillations to LTC_4_ under conditions where both Ca^2+^ entry into, and Ca^2+^ efflux from, the cells were suppressed. This was accomplished by stimulating cells with LTC_4_ in Ca^2+^-free solution supplemented with 1 mM La^3+^, to block plasma membrane Ca^2+^ATPase pumps. Under these conditions, released Ca^2+^ can no longer be exported out of the cell and thus is sequestrated back into the stores [21]. Repetitive Ca^2+^ oscillations are therefore generated in the absence of store-operated Ca^2+^ influx (Fig. 4A; [16]). The number of oscillations declined gradually over time (Fig. 4D), as did the peak amplitude (Fig. 4E). Mitochondrial depolarization (Fig. 4B, D-E) or knockdown of MCU (Figure 4C-E) both accelerated the rundown of the Ca^2+^ oscillations and did so at similar rates. Similar results were obtained when we measured matrix Ca^2+^ directly. Whereas stimulation with LTC_4_ evoked repetitive oscillations in matrix Ca^2+^ when applied in Ca^2+^-free solution containing 1 mM La^3+^ (Figure 4F, I), the matrix rise was significantly reduced by either FCCP exposure (Figure 4G, I) or after knockdown of MCU (Figure 4H, I).

### MCU reduces inactivation of InsP_3_ receptors

An explanation for the accelerated run down of Ca^2+^ oscillations to LTC_4_ in the presence of mitochondrial depolarisation or after knockdown of the MCU is that the loss in mitochondrial Ca^2+^ uptake leads to enhanced Ca^2+^-dependent inactivation of InsP_3_ receptors[23], [24], [25]. To test this idea, we took advantage of the fact that P2Y and cysLT1 receptors target the same intracellular InsP_3_-sensitive Ca^2+^ store in RBL-1 cells. Stimulation with ATP in Ca^2+^-free solution containing 1 mM La^3+^ and FCCP/oligomycin resulted in a single, large Ca^2+^ release transient (Figure 4J). However, the size of this response was reduced considerably when cysLT1 receptors were stimulated first (Figure 4J, K). The size of the Ca^2+^ release transient to ATP in the presence of FCCP/oligomycin was similar to that evoked in the absence of mitochondrial depolarisation (ΔR of 0.44±0.03 and 0.43±0.04, 21 cells each). This is not unexpected, because inactivation of the InsP_3_ receptor can develop slowly, over tens of seconds [26]. The first couple of Ca^2+^ transients to LTC_4_ were also unaffected by FCCP/oligomycin or MCU knockdown (Figure 1E); only subsequent oscillations were smaller and ran down more quickly.

By contrast, the response to thapsigargin was not significantly affected by pre-treatment with LTC_4_ (Figure 4L,M), demonstrating that store Ca^2+^ content was similar for the two conditions. Hence the smaller response to ATP obtained after stimulation with LTC_4_ in cells with depolarised mitochondria would be consistent with the idea that InsP_3_ receptors have inactivated partially following Ca^2+^ release in the presence of impaired mitochondrial Ca^2+^ buffering.

### MCU is required for agonist-evoked gene expression

**Figure 5:**
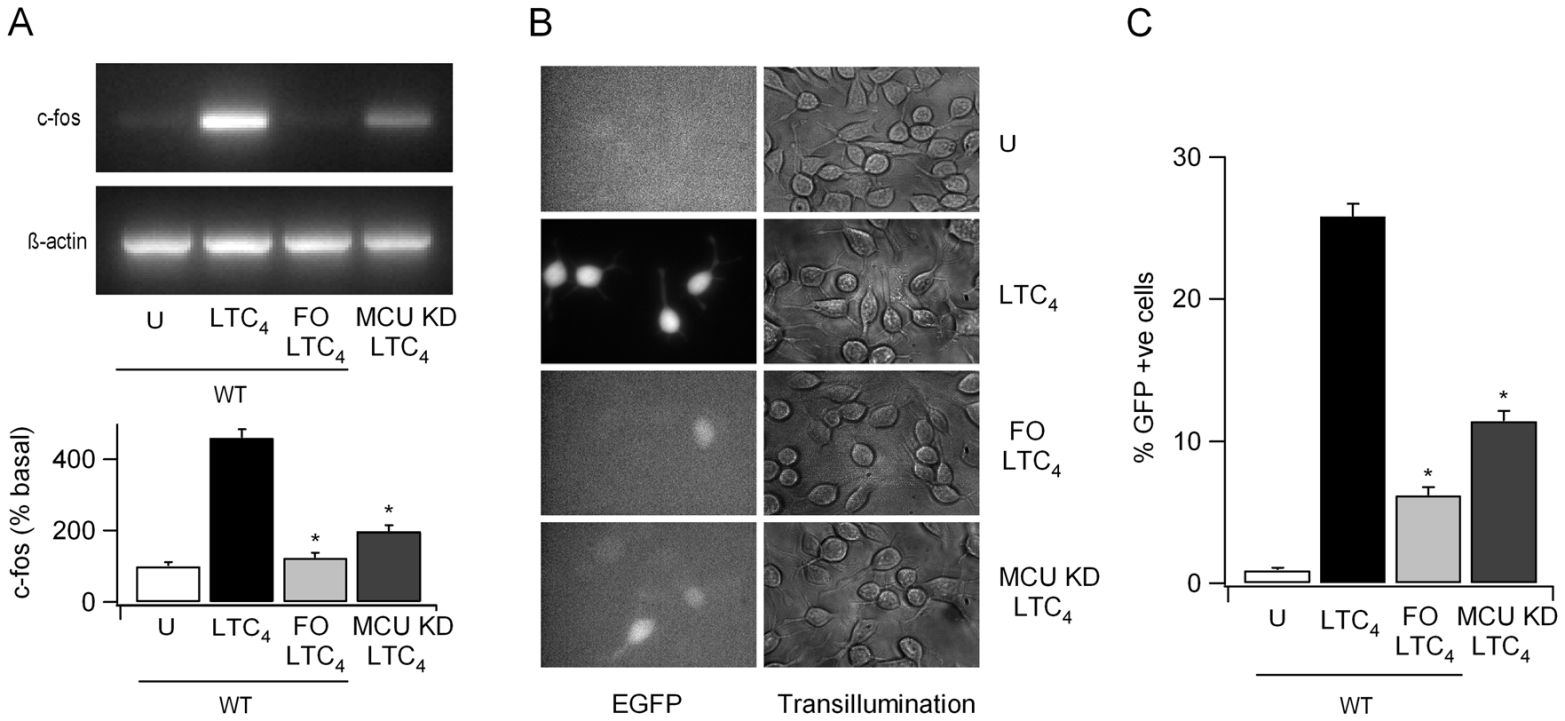
MCU knockdown impairs leukotriene receptor-dependent gene expression. A, Mitochondrial depolarisation or knockdown of MCU suppresses c-fos transcription. Aggregate data (mean of 3 independent experiments) are shown in lower panel. Cells were stimulated with LTC_4_ (160 nM; 4 minutes) in 2 mM external Ca^2+^ and then cells were perfused with Ca^2+^-free solution (without agonist) for a further 41 minutes before RNA extraction. B, Expression of GFP (under an NFAT promoter) is shown for the various conditions indicated. C, Aggregate data from several experiments are compared. In these experiments, cells were stimulated with LTC_4_ in medium for 15 minutes and this was then replaced with LTC_4_-free medium. GFP expression was measured 24 hours later.

We designed experiments to address the functional impact of MCU on Ca^2+^-dependent responses evoked by modest receptor stimulation. Local Ca^2+^ influx through CRAC channels that accompanies oscillatory Ca^2+^ release to cysLT1 receptor activation induces Ca^2+^-dependent gene expression [16], [17], [27]. Stimulation with LTC_4_ increased transcription of the immediate early gene c-fos (Figure 5A) and this was suppressed either by mitochondrial depolarisation (Figure 5A) or after knockdown of MCU (Figure 5A). Ca^2+^ microdomains near open CRAC channels generated during the oscillatory Ca^2+^ signals also activate the transcription factor NFAT. We measured NFAT-driven gene expression by using a GFP reporter gene under an NFAT promoter [17], [28]. Stimulation with LTC_4_ induced a substantial increase in the number of GFP-positive cells (Figure 5B) and this was significantly reduced both by mitochondrial depolarisation (Figure 5B, 5C) or knockdown of MCU (Figure 5B, C).

### MCU sustains store-operated Ca^2+^ entry

**Figure 6:**
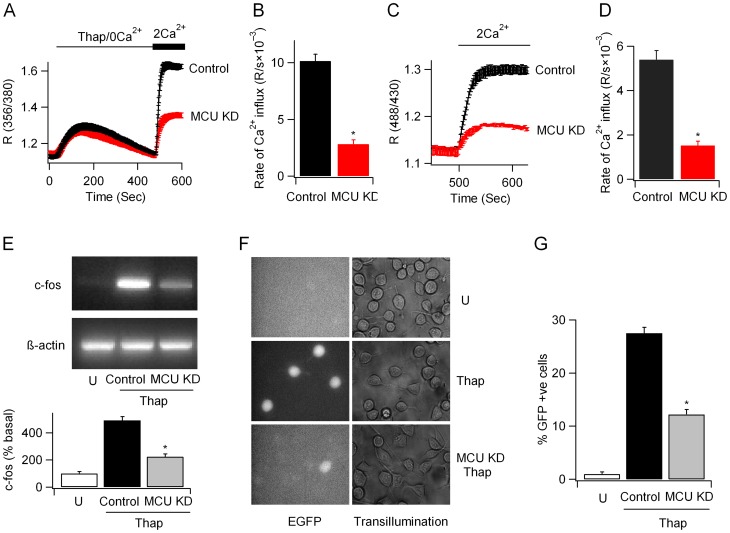
MCU is required for supporting store-operated Ca^2+^ entry. A, Store-operated Ca^2+^ entry, evoked by 2 µM thapsigargin, is inhibited by knockdown of MCU. B, Aggregate data, measuring the rate of rise of cytoplasmic Ca^2+^ following readmission of external Ca^2+^ (as in Panel A) are compared. Control denotes 38 cells and MCU KD 29 cells. C, Matrix Ca^2+^ measurements show that store-operated Ca^2+^ influx is buffered by mitochondria in an MCU-dependent manner. D, Aggregate data from experiments as in Panel C are compared. Control denotes 21 cells and MCU KD 17 cells. E, MCU knockdown reduces c-fos transcription to thapsigargin (2 µM applied for 4 minutes in external Ca^2+^, followed by wash in Ca^2+^-free solution for 41 minutes before RNA extraction). Aggregate data from 3 independent experiments are shown in the lower panel. F, GFP reporter expression (under an NFAT promoter) stimulated by thapsigargin (100 nM) is reduced following MCU knockdown.

Previous work has established that impaired mitochondrial Ca^2+^ buffering, arising from mitochondrial depolarisation, inhibits CRAC channel activity by enhancing Ca^2+^-dependent slow inactivation of the channels [29], [30], [31]. We therefore designed experiments to see if Ca^2+^ uptake by the MCU supported store-operated Ca^2+^ entry. Readmission of external Ca^2+^ to cells 10 minutes after challenge with thapsigargin in Ca^2+^-free solution led to a rapid and large rise in cytoplasmic Ca^2+^ as Ca^2+^ entered through the open CRAC channels (Figure 6A, 6B). Ratiometric pericam experiments revealed that store-operated Ca^2+^ influx was taken up by mitochondria (Figure 6C, 6D). Knockdown of MCU had little effect on thapsigargin-evoked Ca^2+^ release but significantly reduced the rate of rise of cytoplasmic Ca^2+^ due to store-operated Ca^2+^ entry (Figure 6A, B). Mitochondrial Ca^2+^ uptake in response to store-operated Ca^2+^ entry was also impaired by MCU knockdown (Figure 6C, D). The rate of rise of cytoplasmic Ca^2+^ due to store-operated Ca^2+^ entry remained significantly lower after MCU knockdown, compared with control cells when experiments were repeated with elevated external K^+^ (100 mM with a corresponding reduction in Na^+^) to clamp the cell membrane potential close to 0 mV, and thus eliminate potential changes in electrical gradient for Ca^2+^ entry following MCU knockdown. Under these conditions the rate of cytoplasmic Ca^2+^ rise following readmission of external Ca^2+^ (5 mM) to MCU-deficient cells treated with thapsigargin in Ca^2+^-free solution was 28.9±4% that of controls (p<0.01; aggregate data from 21 MCU-deficient cells and 16 control cells).

CRAC channel activation in response to thapsigargin induced robust c-fos expression and this was significantly reduced following MCU knockdown (Figure 6E). Stimulation with thapsigargin also increased expression of the GFP construct under an NFAT protmoter and this too was substantially reduced by MCU knockdown (Figure 6F, G).

## Discussion

The ability of mitochondria to shape the pattern of intracellular Ca^2+^ signals has been documented in numerous cell types (reviewed in [1], [32]). These organelles rapidly take up cytoplasmic Ca^2+^, either that has been released from stores or has entered across the plasma membrane. Ca^2+^ uptake into mitochondria is accomplished through the uniporter, and the pore-forming subunit MCU was recently discovered. The functional importance of MCU was first described in HeLa cells, where knockdown of the protein suppressed mitochondrial Ca^2+^ uptake in response to a maximal dose of the agonist histamine [3], [4]. The MCU is also important for mitochondrial Ca^2+^ uptake in pancreatic beta cells [33], [34] following elevation of cytoplasm Ca^2+^ in response to high concentrations of extracellular glucose. The MCU has also been shown to buffer spontaneous cytoplasmic Ca^2+^ oscillations in cultured neonatal rat cardiac myocytes [35]. These oscillations are believed to reflect Ca^2+^ overload of the sarcoplasmic reticulum. Here, we have addressed three fundamental issues (i) Is the MCU important for regulating cytoplasmic Ca^2+^ signals in response to physiological levels of stimulation? (ii) Through its ability to transport physiological Ca^2+^ pulses, does the MCU influence downstream Ca^2+^-dependent responses such as gene expression? (iii) Is the MCU required to sustain store-operated Ca^2+^ influx?

We have found that cytoplasmic Ca^2+^ oscillations to modest stimulation of cysLT1 receptors are faithfully propagated into mitochondria to generate oscillatory Ca^2+^ signals within the matrix. Knockdown of MCU or a reduction in the electrical gradient for Ca^2+^ flux through the MCU accelerated the run down of these oscillations. Hence the MCU is important for mitochondrial Ca^2+^ uptake in response to physiological levels of cell stimulation. These new findings support and extend our previous patch clamp studies that demonstrated a central role for mitochondria in sustaining CRAC channel activity in the presence of physiological levels of intracellular Ca^2+^ buffering [29], [31], [36]. It has recently been reported that mitochondrial Ca^2+^ uptake is essential for STIM1 aggregation on the ER membrane following store depletion in response to an increase in InsP_3_. According to Deak et al., Ca^2+^ release through InsP_3_ receptors inhibits STIM1 aggregation unless mitochondria are able to buffer the released Ca^2+^. This mechanism could contribute to the accelerated rundown of Ca^2+^ oscillations evoked by LTC_4_ that we have found in the presence of external Ca^2+^ following knockdown of MCU or mitochondrial depolarisation. However, rundown was also prominent after knockdown of MCU in the absence of external Ca^2+^ (Figure 4). Because these latter oscillations are independent of STIM1 and STIM2 [37], impaired aggregation of STIM1 following InsP_3_-dependent Ca^2+^ release is unlikely to account for the faster rundown.

Ca^2+^ oscillations induced by cysLT1 receptor activation in mast cells increase expression of the immediate early gene c-fos, and stimulate calcineurin-dependent dephosphorylation and subsequent nuclear migration of the transcription factor NFAT, both processes occurring in response to local Ca^2+^ influx through CRAC channels that open following the fall in Ca^2+^ within the store during the oscillatory responses [16], [17]. Stimulation of c-fos expression as well as activation of an NFAT reporter gene were both reduced following MCU knockdown or mitochondrial depolarisation. Our data therefore reveal that functional MCU is required for Ca^2+^-dependent gene expression in response to modest receptor activation.

Mechanistically, the run down of the oscillatory Ca^2+^ response that occurred following MCU knockdown or mitochondrial depolarisation was due to impaired Ca^2+^ release rather than compromised Ca^2+^ entry because the response still declined rapidly when cells were stimulated in the absence of external Ca^2+^. Following termination of the oscillatory response, thapsigargin still released Ca^2+^ indicating that the endoplasmic reticulum contained a mobilisable Ca^2+^ pool. InsP_3_ receptors are subject to Ca^2+^-dependent inactivation, a process that inhibits further Ca^2+^ release [25]. Mitochondria are often located close to Ca^2+^ release sites on the endoplasmic reticulum, enabling them to buffer Ca^2+^ microdomains generated by open InsP_3_ receptors [38], [39], [40]. In RBL-1 cells, portions of endoplasmic reticulum are located within 25 nm of mitochondria [41]. By compromising mitochondrial Ca^2+^ buffering, knockdown of MCU or mitochondrial depolarisation would result in a larger local Ca^2+^ rise near active InsP_3_ receptors, leading to strong Ca^2+^-dependent inactivation. Consistent with this, we found that Ca^2+^ release in response to InsP_3_ generated by P2Y receptors was significantly reduced when evoked shortly after run down of Ca^2+^ oscillations in response to leukotriene receptor stimulation.

Our results also show that MCU helps sustain store-operated Ca^2+^ influx. CRAC channels in RBL-1 are subject to inhibition by cytoplasmic Ca^2+^ through two distinct mechanisms. Ca^2+^-dependent fast inactivation is triggered by the build-up of Ca^2+^ microdomains near open channels, develops within milliseconds and is unaffected by mitochondrial Ca^2+^ buffering [29], [42]. Ca^2+^-dependent slow inactivation on the other hand develops over several seconds, requires a rise in bulk Ca^2+^ and is prevented by maintaining mitochondria in an energised state [29], [43]. Slow inactivation is enhanced if mitochondria are depolarised or if the MCU is inhibited with ruthenium red [29]. Our new data strengthen and extend these earlier findings by showing first that mitochondria buffer Ca^2+^ entry through CRAC channels and second that MCU is required to sustain store-operated Ca^2+^ entry. By enabling mitochondria to take up Ca^2+^, MCU sustains CRAC channel activity and downstream gene expression through prevention of the development of Ca^2+^-dependent slow inactivation.

Finally, to our knowledge, our study is the first to demonstrate the importance of MCU in the immune system. Through their ability to buffer cytoplasmic Ca^2+^, mitochondria are important regulators of the spatial and temporal profile of Ca^2+^ signalling in mast cells and T lymphocytes and thereby help determine the extent of activation of important Ca^2+^-driven responses such as secretion of the pro-inflammatory leukotrienes [44] and NFAT activation [17], [30]. Although knockdown of MCU in isolated cells shows a key role for the channel in numerous fundamental physiological processes and functional knockout of the protein impairs gastrulation in zebrafish [45] and bioenergetics in *Trypanosoma* brucei [46], surprisingly the MCU knockout mouse, obtained using the gene trap method, shows only a mild phenotype [47]. The mice are slightly smaller than wild type littermates and are less able to perform strenuous work. The mice also exhibit altered regulation of pyruvate dehydrogenase. On the other hand, human mutations of MICU1 are associated with proximal myopathy, learning difficulties and a progressive extrapyramidal movement disorder and which are thought to arise from defective mitochondrial Ca^2+^ signaling [48]. Future work, using conditional knock out of MCU in immune cells, will help shed insight into the role of the channel in the immune response.

## Methods

### Cell culture

RBL-1 cells were purchased from ATCC (via UK supplier LGC) and were cultured at 37°C with 5% CO_2_ in Dulbecco's modified Eagle's medium (DMEM) supplemented with 10% fetal bovine serum and 1% penicillin/streptomycin, as described [17]. Cells were split using Trypsin-EDTA and plated onto glass coverslips for use 24–48 hours later.

### Fluorescence Ca^2+^ measurements

Cytosolic Ca^2+^ measurements were carried out at room temperature using the IMAGO charge-coupled device camera-based system from TILL Photonics, as described previously [44]. Cells were alternately excited at 356 and 380 nm (20-ms exposures), at 0.5 Hz. Images were analyzed offline using IGOR Pro for Windows. Cells were loaded with Fura-2/AM (1 µM) for 40 min at room temperature in the dark and then washed three times in standard external solution composed of 145 mM NaCl, 2.8 mM KCl, 2 mM CaCl_2_, 2 mM MgCl_2_, 10 mM D-glucose, 10 mM HEPES, pH 7.4, with NaOH. Cells were left for 15 min to allow further de-esterification. Ca^2+^-free solution had the following composition: 145 mM NaCl, 2.8 mM KCl, 2 mM MgCl_2_, 10 mM D-glucose, 10 mM HEPES, 0.1 mM EGTA, pH 7.4, with NaOH. Ca^2+^-free solution containing La^3+^ had the following composition: 145 mM NaCl, 2.8 mM KCl, 2 mM MgCl_2_, 10 mM D-glucose, 10 mM HEPES, 1 mM LaCl_3_, pH 7.4, with NaOH. Ca^2+^ signals are plotted as R, which denotes the 356/380 nm ratio. Rmin was 0.42 and Rmax was 2.1. LTC_4_ was bought from Cayman Chemicals.

### Mitochondrial Ca^2+^ measurements

Cells expressing the mitochondrial ratiometric pericam were analyzed 24 hours after transfection by videoimaging using the TiLL Photonics system. Cells were illuminated alternately at 430 and 488 nm (20 msec exposures) and the emitted light was filtered at >510 nm.

### Measurement of mitochondrial membrane potential

Cells were loaded with TMRE (50 nM) in standard external solution for 30 minutes in the dark, followed by three washes in external solution. Cells were excited at 545 nm and emitted light was collected at >560 nm.

### Gene reporter assay

24–36 hours following transfection with the EGFP-based reporter plasmid that contained an NFAT promoter (gift from Dr Yuri Usachev, University of Iowa), cells were stimulated with LTC_4_ and the % of cells expressing EGFP measured subsequently (∼24 hours later). Gene expression was defined as fluorescence 3xSD> cell autofluorescence, measured in non-transfected cells, as described [49]. Cells were stimulated in culture medium and maintained in the incubator for ∼24 hours prior to detection of EGFP. In experiments were thapsigargin was the stimulus, cells were exposed to 100 nM thapsigargin for 15 minutes in culture medium before thapsigargin-containing medium was replaced with normal DMEM overnight.

### siRNA knockdown

Cells were transfected with the Amaxa system, as described. siRNA against MCU was from Origene (Cat No.: SR508660).

### RT-PCR

Total RNA was extracted from RBL cells by using an RNeasy Mini Kit (Qiagen), as described [18]. RNA was quantified spectrophotometrically by absorbance at 260 nm. Total RNA (1 µg) was reverse-transcribed using the iScriptTM cDNA Synthesis Kit (Bio-Rad), according to the manufacturer's instructions. Following cDNA synthesis, PCR amplification was then performed using BIOX-ACTTM. ShortDNAPolymerase (Bioline) with primers specific for the detection of c-fos were synthesized by Invitrogen. The PCR products were electrophoresed through an agarose gel and visualized by ethidium bromide staining.
